# Significance of Input Correlations in Striatal Function

**DOI:** 10.1371/journal.pcbi.1002254

**Published:** 2011-11-17

**Authors:** Man Yi Yim, Ad Aertsen, Arvind Kumar

**Affiliations:** Bernstein Center Freiburg and Neurobiology & Biophysics, Faculty of Biology, University of Freiburg, Freiburg, Germany; Indiana University, United States of America

## Abstract

The striatum is the main input station of the basal ganglia and is strongly associated with motor and cognitive functions. Anatomical evidence suggests that individual striatal neurons are unlikely to share their inputs from the cortex. Using a biologically realistic large-scale network model of striatum and cortico-striatal projections, we provide a functional interpretation of the special anatomical structure of these projections. Specifically, we show that weak pairwise correlation within the pool of inputs to individual striatal neurons enhances the saliency of signal representation in the striatum. By contrast, correlations among the input pools of different striatal neurons render the signal representation less distinct from background activity. We suggest that for the network architecture of the striatum, there is a preferred cortico-striatal input configuration for optimal signal representation. It is further enhanced by the low-rate asynchronous background activity in striatum, supported by the balance between feedforward and feedback inhibitions in the striatal network. Thus, an appropriate combination of rates and correlations in the striatal input sets the stage for action selection presumably implemented in the basal ganglia.

## Author Summary

The striatum is the main input station of the basal ganglia and plays a crucial role in multiple motor and cognitive functions. Striatum is a recurrently connected network of GABAergic medium spiny neurons (MSNs), which receive strong feedforward inhibition from the fast spiking interneurons and massive excitatory afferents from various regions of the neocortex via the cortico-striatal projection neurons. Here, we study the effects of input rate and temporal correlations on signal representation in a computational model of striatum. We show that when individual striatal neurons receive weakly correlated input from the neocortex, signal representation is enhanced. Surprisingly, though, if the inputs to two striatal neurons are correlated, signal representation is impaired. In a restricted sense, correlation in the inputs to two neurons implies that these neurons share their input, which according to our model would not be optimal for signal representation. Interestingly, cortico-striatal projections are structured in such a way that neighboring MSNs are not likely to share their presynaptic cortical neurons. Thus, we suggest that an appropriate structure of correlations in the striatal inputs sets the stage for implementation of various tasks performed by the basal ganglia, supported by the special anatomical structure of the cortico-striatal projections.

## Introduction

The striatum is the main input stage of the basal ganglia and plays an important role in various cognitive and motor functions [1]–[5]. With its involvement in multiple behavioral tasks, the computational role of the striatum is of crucial interest. The presence of recurrent inhibitory projections among the main constituent cells, the medium spiny neurons (MSNs) led to the suggestion that the Winner-Take-All (WTA) dynamics presents the main working principle of the striatum [6], [7]. However, experimental evidence of low connection probability among MSNs and weak recurrent inhibitory synapses [8]–[11] suggest that the neural hardware in the striatum cannot support such WTA dynamics. Thus, Ponzi and Wickens (2010) recently argued for a ‘winner-less-competition’ based on hypothesized cell assemblies in the ongoing striatal network activity.

In most computational theories of striatum function, much emphasis is put on the connectivity of the striatal network and the individual neuron properties. Interestingly, though, the connectivity pattern of the cortico-striatal input projections is mostly ignored. Anatomical evidence suggests that these input projections are structured in a special manner. Each striatal neuron receives massive synaptic input from the cortex. Moreover, individual cortical locations give rise to multiple separate foci of innervation in the striatum, with axons from functionally related cortical regions sharing common focal striatal innervation zones [12], [13]. Therefore, striatal neurons are expected to share their cortical presynaptic pools to a considerable degree. Surprisingly, though, the sharing of inputs between neighboring striatal neurons is estimated to be relatively small [12], [13]. However, because task related cortical activity is modulated in both correlation and firing rate [14]–[18], individual striatal neurons are indeed expected to receive correlated inputs.

Thus, to understand the computational role of the striatum in different behavioral tasks, it is of key importance to understand how the spatio-temporal structure of the input correlations can influence the striatal response and, hence, striatal function. Therefore, here, we investigate the functional consequences of input correlations on the representation of cortical activity in the striatum. We show that weak correlation in the inputs to individual neurons enhances the saliency of the signal representation. Interestingly, the striatal response to cortical input is most salient when striatal neurons do not share their inputs. Thus, sharing of inputs among striatal neurons degrades the signal representation.

In summary, we suggest a functional role for the special anatomy of cortico-striatal projections by ensuring that individual striatal neurons are less likely to share their cortical inputs, while at the same time they each receive weakly correlated inputs. Preliminary results were previously presented in abstract form [19].

## Results

**Figure 1:**
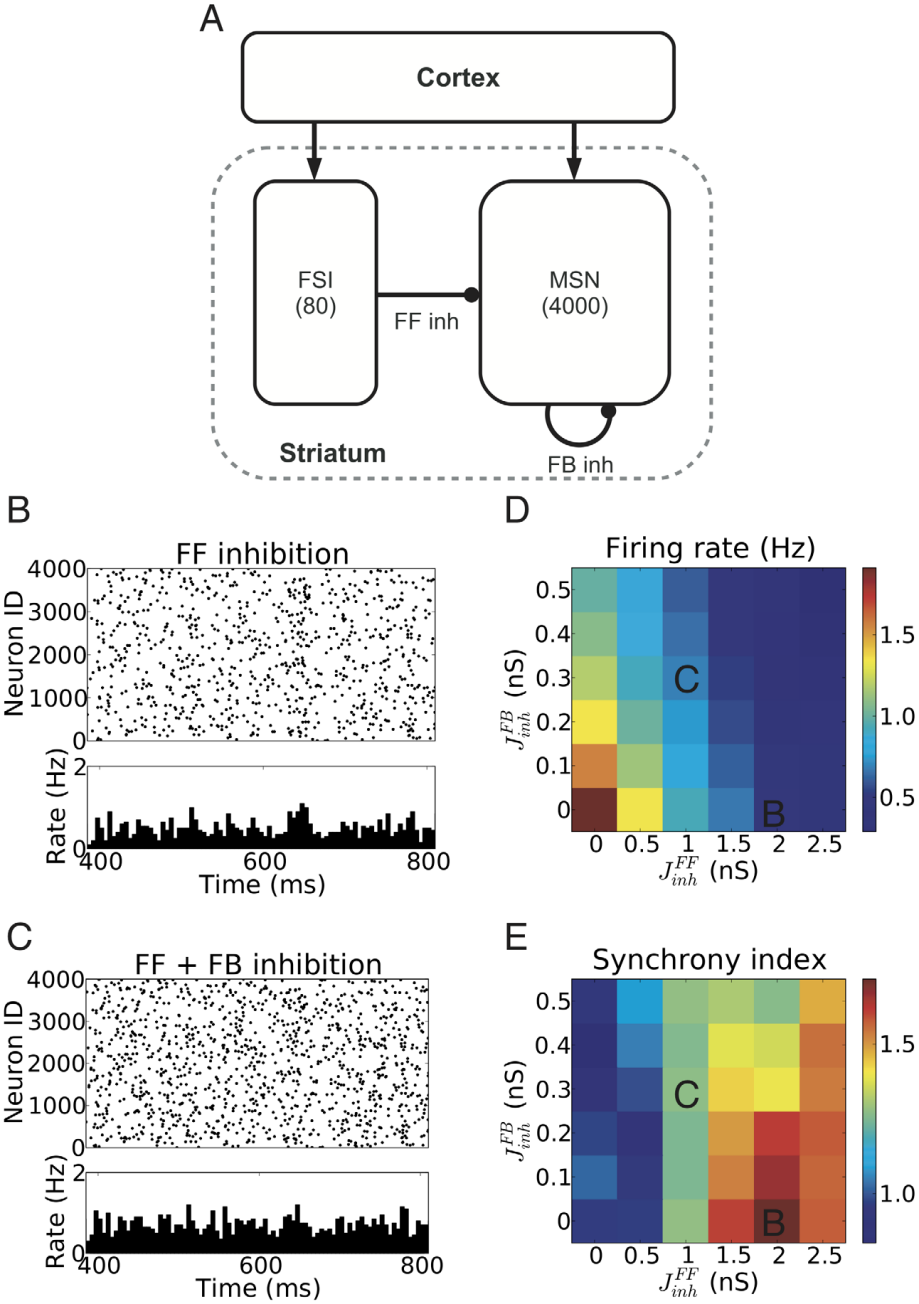
Different activity states in a striatum network model. (A) Schematic microcircuit of the striatum. (B) Raster plot of MSNs spiking activity in a striatum network model dominated by FF inhibition. The FF inhibition induced synchrony, but not oscillations in the MSN population activity. (C) Low firing rate and asynchronous, irregular spiking activity in a striatum network in presence of both FF and FB inhibitions. (D) Mean firing rate of the MSNs for different feedforward (FF) and feedback (FB) inhibition strengths. (E) Synchrony index (cf. Methods) of the population activity for different FF and FB inhibition strengths.

The striatum is a recurrent inhibitory network driven by excitatory projections from the cortex (Fig. 1A). Such networks have been extensively studied for their synchronization and oscillatory properties [20]–[23]. The striatum network, however, differs from the standard recurrent inhibitory network in that the FF and FB inhibition are clearly segregated, because the MSNs do not project to the FSIs. FF inhibition can alter the effective integration time in postsynaptic neurons [24] and, thus, may influence synchrony and propagation of activity in neuronal networks [25]. Likewise, FB inhibition alone in a recurrent network can induce fast oscillations and network synchronization [21], [26]. Therefore, to understand the dynamics of a striatum-type network, we first investigated the role of both FF and FB inhibition in shaping the global network dynamics of the striatum.

### Dynamical states of ongoing activity in the striatum

In the striatum, FSIs make divergent projections with strong synapses onto the MSNs. Because the MSNs outnumber the FSIs by far, this projection scheme results in a highly correlated FF inhibition as a consequence of sharing presynaptic FSIs. Therefore, in a scenario where FF inhibition is dominant, high inhibitory input correlations may synchronize the MSN population activity (Fig. 1B). Likewise, dominant FB inhibition, because of its recurrent nature, may also induce synchrony in the MSN population [21]. We found, however, that within the biologically realistic parameter range, FB inhibition in the striatum was not strong enough to induce oscillations (data not shown).

However, FB inhibition could impair the synchrony induced by the FF inhibition (Fig. 1C). To further investigate this joint effect of FB and FF inhibition on network synchrony, we systematically varied the strength of the two modes of inhibition independently (Figs. 1D, E). We found that for FF and FB inhibition both weak, striatum activity remained asynchronous. Strong FF inhibition induced synchrony in the network, which could be reduced by an increase in FB inhibition (Fig. 1E). For biologically realistic ranges of FF and FB inhibition strengths [10], which ensured low firing rates in the striatum network, we observed only weak synchrony in the ongoing network activity.

In the healthy striatum, the firing rates of MSNs can vary between 0.2 Hz and 20 Hz [27], [28], depending on the behavioral state of the animal, while in the quiet awake state, most studies reported MSN firing rates to be less than 2 Hz. At the same time, there is no clear experimental evidence for synchrony and oscillations in the striatum during ongoing activity. Nevertheless, some experimental studies in behaving monkeys reported phase locking of a fraction of recorded putative MSNs to 10–25 Hz oscillations in local field potentials (LFP) [29]. Note that such phase locking of single-neuron spikes to LFP oscillations does not necessarily imply (or require) synchronization of population spiking activity. Thus, the observed low firing rates (Fig. 1D) and weak synchrony (Fig. 1E) in the presence of both FB and FF inhibition in our network model are consistent with the *in vivo* ongoing activity recorded in the striatum of healthy animals [30].

In our network simulations, multiple combinations of FB and FF inhibition could generate a biologically realistic baseline activity in the striatum network (Figs. 1D, E). Thus, to further investigate the representation of cortical inputs in striatum network activity, we adjusted the network parameters to obtain a near-asynchronous activity state (synchrony index 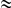
1.28) at low firing rate (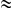
0.7 Hz). These settings were applied in all subsequent sections, unless otherwise indicated.

### Effect of input correlations on the striatum response

There is ample experimental evidence for an increase in firing rates [31]–[34] and the emergence of correlations [14]–[18] in stimulus or task related cortical activity. Thus, at least during a behavioral task, the striatum is likely to receive cortical activity with modulations of firing rates and correlations. Therefore, to understand the representation of task-related cortical activity in the striatum, we modeled the cortical stimulus related activity as a MIP (multiple interacting process) type ensemble of correlated Poisson spike trains [35]. We chose this model of ensemble spiking activity because (1) it can be formulated in analytical terms and has been studied in great detail [35], [36] and (2) it allows for systematic and independent variations of firing rates and pairwise correlations.

**Figure 2:**
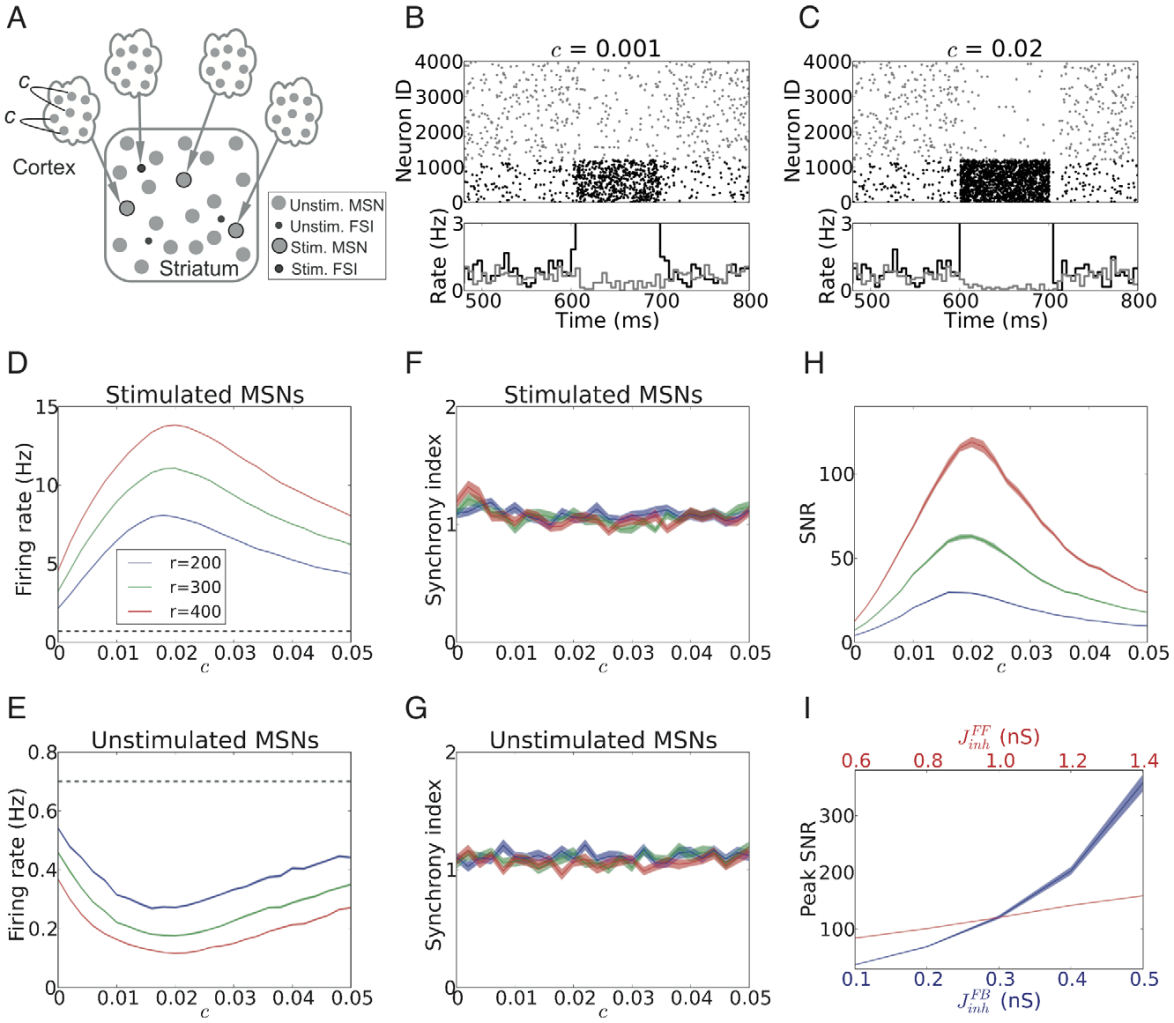
Signal representation in the striatum network when stimulus input to individual stimulated neurons was correlated. (A) Scheme of stimulus configuration-I (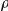 = 0; 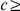
0) presented to a fraction of striatum neurons, on top of the background excitatory input from the cortex and background inhibitory input from other striatal neurons. (B,C) Examples of MSNs spiking responses when 30% of striatal neurons were stimulated for 100 ms (starting at 600 ms) with excitatory input with an ensemble firing rate 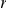 = 400 Hz and low (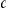 = 0.001; B) or high (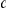 = 0.02; C) input correlations, respectively. (D,E) Firing rate of the stimulated MSNs (D) and the unstimulated MSNs (E), averaged over the stimulation epoch, as a function of input correlation 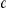, for two different input firing rates. Observe that the response rate in both subpopulation varied in a non-monotonic fashion with increasing input correlation 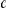. The dashed line indicates the level of baseline activity. (F,G) Synchrony index of the stimulated MSNs (F) and the unstimulated MSNs (G) as a function of input correlation 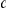. The synchrony index of the stimulated MSNs is close to 1 so there is no significant synchrony. (H) Signal-to-noise ratio (SNR) of the striatum network, quantified by the ratio of the average firing rates of the stimulated and unstimulated MSNs, as a function of input correlation 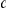. Observe that SNR varied in a non-monotonic fashion with increasing input correlation 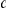. By contrast, SNR increased monotonically with input firing rate (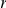). (I) Peak SNR of the striatum network as a function of input correlation 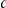 for different strengths of feedback 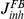 and feedforward inhibition 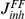. The blue trace shows peak SNR for different values of 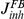 and a fixed 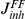 = 1 nS. The red trace shows peak SNR for different values of 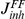 and a fixed 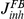 = 0.3 nS. Observe that increasing either type of inhibition increased the peak SNR, because stronger inhibition is more effective in suppressing the background activity.

**Figure 3:**
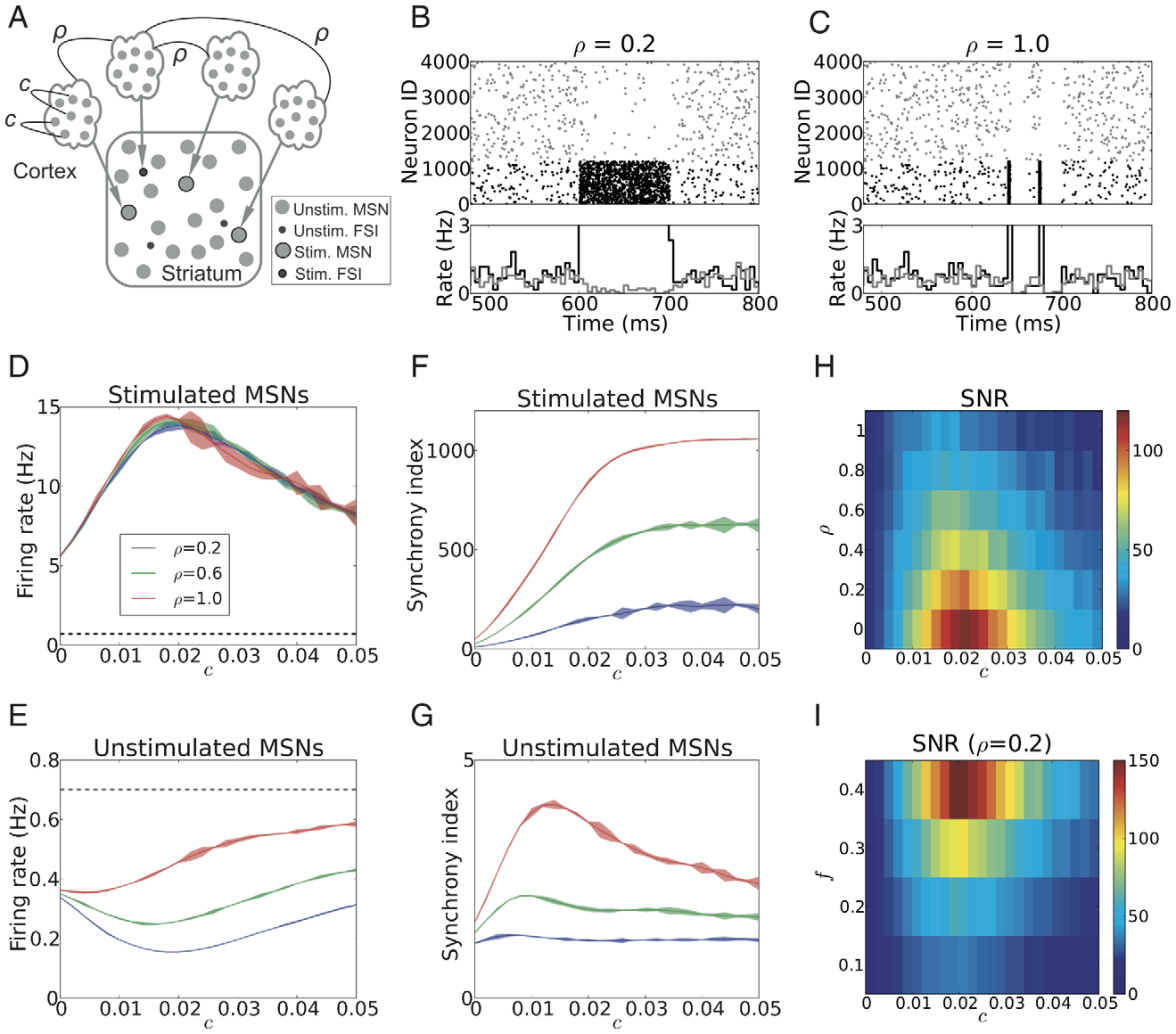
Signal representation in the striatum network when stimulated neurons received correlated inputs. (A) Scheme of stimulus configuration-II (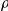 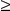
0; 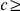 0) presented to a fraction of striatum neurons, on top of the background excitatory input from the cortex and background inhibitory input from other striatal neurons. (B,C) Examples of MSNs spiking responses for two different stimuli to 30% of striatal neurons, with identical input firing rate (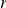 = 400 Hz) and internal correlation (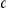 = 0.02), but different shared correlations across stimulated neurons: low (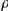 = 0.2; B) or high (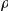 = 1.0; C), respectively. (D,E) Firing rate of the stimulated MSNs (D) and the unstimulated MSNs (E), averaged over the stimulation epoch, as a function of input correlation 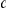, for three different values of shared correlation 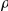. (F) Synchrony index of the stimulated MSNs (F) and the unstimulated MSNs (G) as a function of input correlation 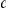, for three different values of shared input correlation 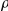. Observe that the synchrony index of the stimulated MSNs increased both with increasing 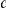 and increasing 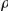, due to the fact that larger 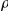 led to more shared inputs among stimulated neurons, while larger 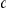 resulted in more reliable spiking. In the unstimulated MSNs, (low values of 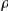 did not influence synchrony, whereas high 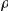 increased synchrony, due to the synchronized inhibition, induced by the synchronized spiking of the stimulated population. (H) Signal-to-noise ratio (SNR) of the striatum network as a function of both input correlation 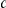 and shared correlation 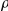 at 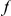 = 0.3. Observe that maximal SNR was obtained for weakly correlated (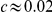) input to individual striatal neurons and uncorrelated (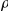 = 0) inputs to different striatal neurons. (I) Signal-to-noise ratio (SNR) of the striatum network as a function of both input correlation 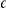 and stimulated fraction 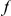 at 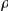 = 0.2. Observe that maximal SNR was obtained for weakly correlated (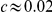) input to a larger fraction of stimulated MSNs (larger 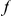).

To systematically investigate the effects of input correlations on the striatal response, we considered two input configurations. In the input configuration-I, each stimulated neuron in the striatum received MIP type activity with an input correlation 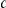, while the inputs to different striatum neurons remained uncorrelated (Fig. 2A). This input configuration refers to a scenario in which striatum neurons do not share their presynaptic pools (cf. Methods). In the input configuration-II, we introduced additional correlation between the inputs of different stimulated neurons (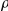), while each of them still received MIP type input with correlation 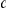 (Fig. 3A). When 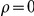, this input configuration is identical to the configuration-I. 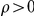 refers to a scenario in which either the striatum neurons shared their presynaptic pools or the presynaptic pools of different striatal neurons were themselves correlated (cf. Methods).

To quantify the signal representation of the striatum in both input configurations, we measured the signal-to-noise ratio (SNR, cf. Methods) in each case. Here, we are interested in the statistical properties of the stimulus (input firing rate, correlations 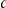 and 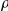) that maximize the SNR in the striatum.

#### Input configuration - I: Correlated input to individual neurons

Figs. 2B, C show the responses of MSNs when 30% of striatal neurons were stimulated [37] for 100 ms (starting at 600 ms) by an excitatory input with an ensemble firing rate of 400 Hz, but different correlations 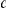. Higher input correlation (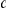 = 0.025, Fig. 2C) resulted in a higher firing rate in the stimulated neurons and a lower rate in the unstimulated neurons, compared to the case of lower input correlation (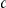 = 0.001, Fig. 2B).

The effect of input correlation 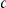 on MSN population activity at different input firing rates is summarized in Figs. 2D–H. Consistent with previous findings [35], the output firing rate of the stimulated neurons varied in a non-monotonic fashion with increasing input correlation 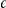. Thus, for a given input firing rate, there existed an optimal value of input correlation (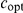), that maximized the output rate of the stimulated neurons and, at the same time, minimized the activity of the unstimulated neurons (Fig. 2E). For input correlations below 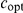, the average number of spikes in coincidence clusters in the input was smaller than the average number required to make a postsynaptic neuron spike, thereby limiting the output rate of the stimulated neurons and, hence, the suppression of the unstimulated neurons. By contrast, for input correlations beyond 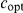, the average number of spikes in coincidence clusters in the input was larger than the average number required to make a postsynaptic neuron spike. In that case, input spikes were effectively being wasted, causing such higher input correlation to result in a smaller response in the stimulated neurons and, hence, a smaller suppression of the activity of unstimulated neurons. In between, at the optimal input correlation (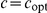), the SNR was maximized as shown in Fig. 2H. An increase in input firing rate always increased the SNR, however, the non-monotonicity of SNR as a function of 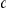 remained, with the peak continuing to be at 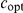.

Note that, without an external stimulus the network is in an asynchronous state, due to the coexistence of FB and FF inhibitions (Fig. 1E). The presentation of an excitatory stimulus did not cause any synchrony in the network, neither in the stimulated (Fig. 2F), nor in the unstimulated neurons (Fig. 2G).

In summary, the above results indicate that input correlations of strength around 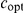 are most effective in enhancing the SNR in the striatum.

Finally, we note that increasing the strength of either FB or FF inhibition increased the peak SNR in our model (Fig. 2I). Similar results were obtained for the SNR outside the range of optimal input correlation, i.e. when 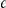 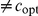 (data not shown). Evidently, an increase in inhibition in the network (either FF or FB inhibition) reduced the response of both stimulated and unstimulated neurons, compared to the low inhibition state. Nevertheless, the activity of the unstimulated neurons was more strongly suppressed, resulting in an increased SNR.

#### Input configuration - II: Shared correlation among inputs to different neurons

Next, we allowed for correlation among the inputs to the stimulated neurons (cf. Methods; Fig. 3A) and investigated the effects of such shared input correlation (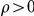) on the SNR in the striatum.

Figs 3B and C show two examples of striatum activity when 30% of neurons were stimulated with an external excitatory input with an ensemble firing rate of 400 Hz and internal correlation 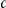 = 0.02, but with different shared input correlations 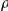. Low shared correlation (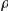 = 0.2, Fig. 3B) resulted in an increase in the firing rate of the stimulated neurons, and a corresponding decrease in activity of the unstimulated neurons. By contrast, a high shared correlation (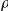 = 1.0, Fig. 3C) induced strong intermittent (Poisson distributed) synchronous spike clusters in the stimulated neurons. Each such synchronous event in the stimulated neurons strongly inhibited the activity of the unstimulated neurons for a short time; in between such events, the unstimulated neurons' activity returned to baseline level. Thus, the unstimulated neurons were inhibited repeatedly, but only for short intervals after the onset of stimulation, eventually resulting in a relatively small decrease in their average activity. As a result, SNR was smaller for 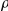 = 1 than for 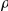 = 0.2, and, in fact, as we will see later (Fig. 3H), even smaller than for 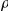 = 0.

To quantify the effect of both types of input correlations on the SNR of the striatal network, we systematically and independently varied both the correlation within (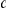) and between (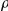) the input pools to stimulated MSNs (Figs. 3D–H).

Because the activity statistics of the input to individual stimulated neurons was independent of the shared input correlation 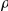, the non-monotonic relationship between the output firing rate of the stimulated MSNs and input correlation 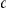 remained unaffected (Fig. 3D, compare Fig. 2D). By contrast, 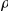 strongly affected the suppression of the activity of the unstimulated neurons, with an increase in 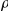 leading to a reduction in suppression (Fig. 3E). Furthermore, an increase in 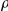 reduced the value of 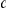 for which maximal suppression of the activity of the unstimulated neurons could be obtained (Fig. 3E).

Why did the shared input correlation 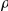 influence the unstimulated neurons and not the stimulated neurons? To understand this, we measured the synchrony of the activity in the stimulated and unstimulated neurons. We found that 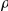 influenced the synchrony in the network in a complex manner. First, for 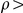
0, the synchrony in the stimulated neural population monotonically increased with 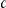 (Fig. 3F), in strong contrast with the observations made in the absence of shared input correlations (input configuration - I; cf. Fig. 2F). An increase in 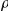 further enhanced this synchrony, with the maximum reached for 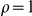, when all stimulated neurons received identical stimulus inputs. Second, for low values of shared correlation 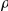, the input correlation 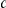 did not influence the synchrony in unstimulated neurons. However, increasing 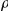 introduced synchrony in the unstimulated MSNs, weak still for smaller 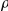, but stronger for larger 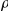 and an increasing tendency for a non-monotonic dependence on 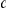, peaking around 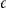 = 0.01 (Fig. 3G).

**Figure 4:**
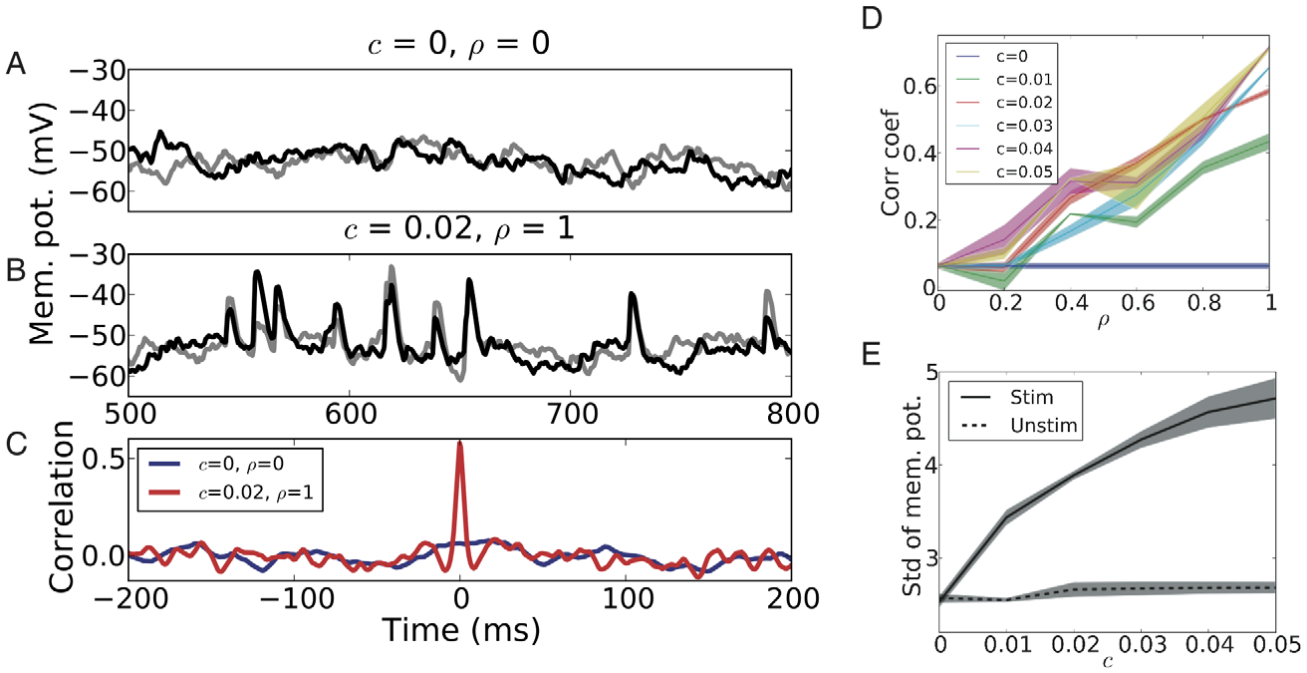
Correlations of the free membrane potentials of stimulated MSNs in the striatum network. (A) Example of the free membrane potential traces of two stimulated MSNs without any input correlation (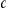 = 0, 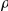 = 0). As there was no correlation, neither within nor between the inputs to the two MSNs, the free membrane potentials did not show any significant correlation. (B) Example of the free membrane potential traces of two stimulated MSNs with moderate individual input correlation (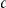 = 0.02) and full shared correlation (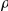 = 1). Since here, the two MSNs were driven by correlated synaptic inputs, the free membrane potential traces showed significant correlation, together with substantial membrane potential fluctuations. (C) Cross correlation of the free membrane potentials of stimulated MSN pairs as a function of time lag 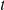 for the two stimulus protocols. Strong correlation was observed at zero time lag for moderate individual input correlation (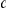 = 0.02) and maximum shared input correlation (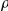 = 1). (D) Average correlation coefficient at zero time lag of the free membrane potentials of stimulated MSN pairs as a function of shared input correlation 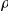, for different values of individual input correlation 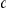. The correlation coefficient increased monotonically with 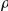 and did not show any dependence on 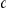. Note that the correlation coefficient had a small positive value for 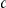 = 0, because of the small synchrony effect arising from the FB and FF inhibitions. (E) Standard deviation of free membrane potential fluctuations for stimulated (solid lines) and unstimulated (dashed lines) MSNs as a function of individual input correlation 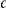 at different values of 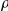. The free membrane potential fluctuation of the stimulated MSNs increased monotonically with 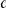, whereas that of the unstimulated MSNs did not show any significant change.

These various effects of 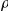 and 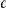 could also be seen in the subthreshold activities of the neurons (Fig. 4A, B). Thus, we measured the cross-correlation between free membrane potentials (cf. Methods) of the stimulated striatal neurons. Consistent with our expectations, the zero time lag cross-correlations (Fig. 4C) between the subthreshold activities of the stimulated neurons increased with 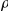 (Fig. 4D), independently of the value of 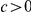. Only in the limiting case of no input correlation (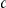 = 0), the subthreshold activities showed no correlation whatsoever, independently of 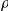, of course. The effect of increasing 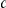 was more clearly visible in the size of the membrane potential fluctuations, characterized by the standard deviation, which monotonically increased with 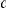 (Fig. 4E). Larger 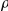 resulted in slightly smaller membrane potential fluctuations in the stimulated neurons. Subthreshold activity correlations between unstimulated neurons or between stimulated and unstimulated neurons were highly variable (data not shown) and depended strongly on the connectivity. More detailed analyses, explicitly taking into account the connectivity among the neurons, are needed to understand this high variability. The unique way (Figs. 4D,E) in which the two descriptors (pairwise correlation and size of membrane potential fluctuations) of the stimulated neurons reflect the correlation structure (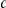,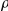) of the stimulus input to the network suggests an interesting novel application for analyzing data recorded in experiments in behavioral tasks. Analyzing the behavior of these two descriptors for neurons which increased their activity during a task (therefore putatively being stimulated neurons) could potentially provide the means to determine, by ‘reverse engineering’, the correlation state (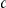,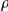) of the cortical input to the striatum during the task.

Next we quantified the SNR in the presence of shared input correlation 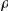. Fig. 3H shows the SNR as a function of 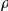 and 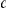. Evidently, 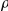 did not influence the non-monotonic nature of the SNR as a function of 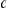. However, the maximal SNR decreased monotonically as a function of 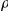 (Fig. 3H), due to the reduced supression of the unstimulated neurons.

The proportion of neurons responding to a stimulus can change in different learning stages [2], [28], [37]. Therefore, we characterized the effects of varying the fraction of stimulated MSNs (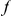) on the signal representation. Fig. 3I shows the SNR as a function of 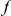 and 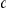. Since a larger fraction of stimulated MSNs 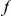 implied an increased inhibition leading to a stronger suppression of the unstimulated MSNs activity, the SNR increased monotonically with an increase in 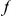.

In summary, therefore, the outcome of our analysis (Fig. 3H) suggests that to maximize the signal representation in the striatum, the input to individual striatal neurons should preferably be weakly correlated (i.e. 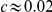 in our model), whereas different striatal neurons should preferably receive uncorrelated inputs (i.e. 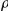 = 0 in our model). This finding is interesting, because anatomical evidence suggests that neighboring MSNs are not likely to share their inputs [13] and afferents arriving at an innervation zone originate from functionally related brain regions [12], suggesting that 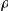 is likely to be very small, if not zero and 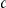 is likely to be finite and not zero.

### Correlated feedforward inhibition

**Figure 5:**
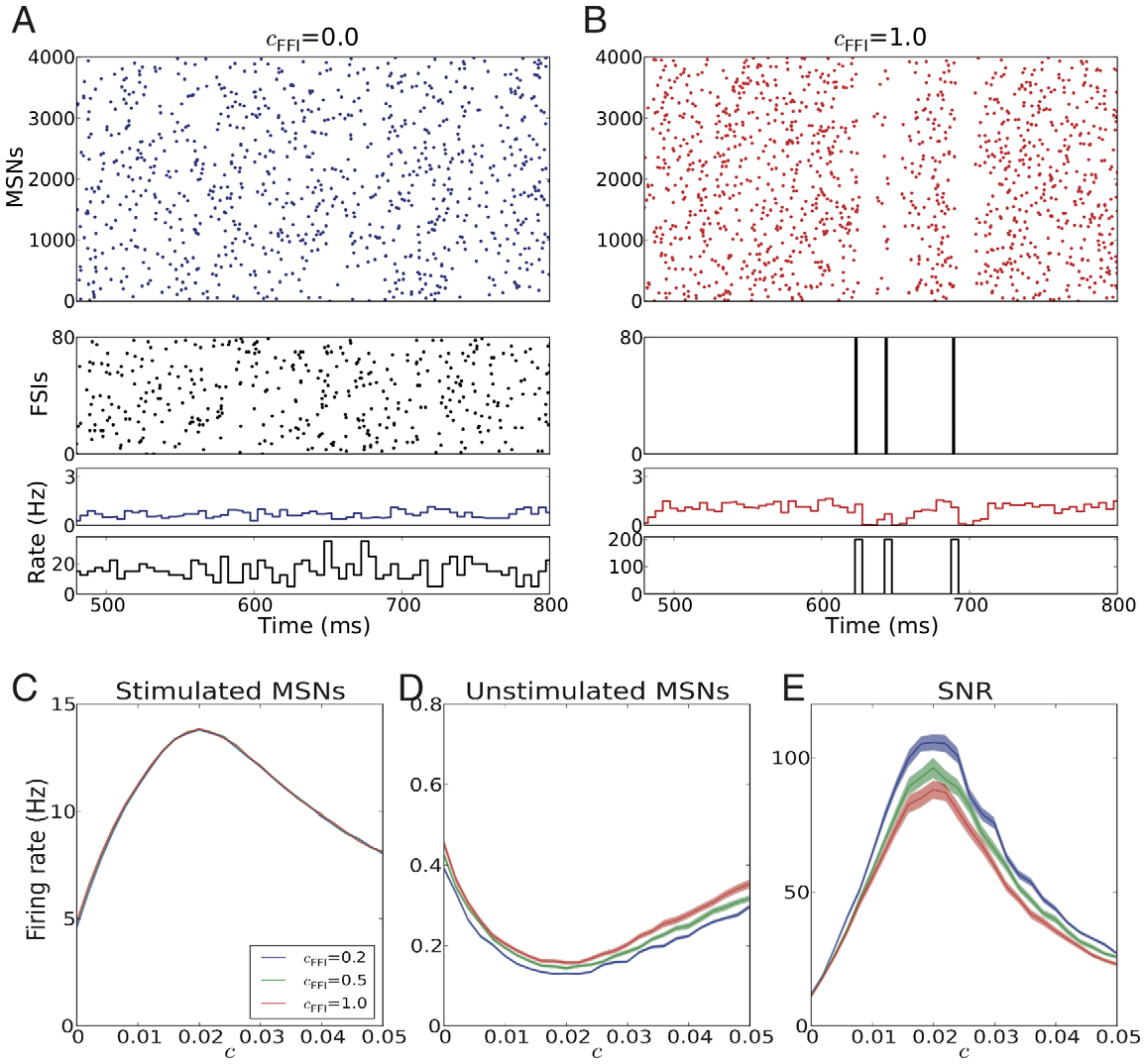
Network dynamics and signal representation in the striatum network when the spiking of FSIs is correlated. (A) Spiking activity in the striatum for 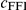 = 0. Blue and black rasters show the spiking activity of MSNs and FSIs, respectively. PSTHs of the corresponding rasters are shown at the bottom. (B) Spiking activity in the striatum for 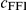 = 1. Red and black rasters show the spiking activity of MSNs and FSIs, respectively. PSTHs of the corresponding rasters are shown at the bottom. (C) Firing rate of the stimulated MSNs, averaged over the stimulation epoch, as a function of input correlation 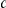, for four different values of 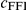. (D) Firing rate of the unstimulated MSNs, averaged over the stimulation epoch, as a function of input correlation 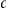, for four different values of 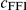. (E) Signal-to-noise ratio (SNR) of the striatum network as a function of input correlation 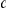, for four different values of FSIs' output correlation 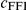. Observe that inhibitory input correlation decreased the SNR, which maintained its non-monotonicity and shifted its peak to a slightly lower value of 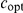.

Both chemical synapses and gap junctions are present among striatal FSIs. Experimental data as well as network simulations suggest that gap junctions can cause global synchrony [38]. While there is no strong evidence for synchronization of striatal FSIs due to gap junctions, neither from experiments [27], nor from modeling studies [39], it is nevertheless of interest to understand the effect of FFI correlations (irrespective of whether they are mediated by gap junctions or chemical synapses or whether they are input driven) on the firing pattern of MSNs and the signal representation in the striatum. We observed that when the FSI spiking activities were uncorrelated, the MSNs received a largely stationary feedforward inhibition, which was reflected in equally stationary firing rates of the MSNs (Fig. 5A). By contrast, when FSI activity was correlated, large intermittent fluctuations in the FF inhibition caused the MSNs to be repeatedly inhibited for short epochs at irregular intervals (e.g. Fig. 5B for 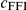 = 1).

When a fraction of MSNs received extra cortical input, correlated FF inhibition resulted in an increased firing rate in the stimulated MSNs for small within-pool correlation (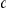). For larger within-pool correlation, 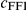 did not influence the activity of the stimulated MSNs (Fig. 5C). On the other hand, in the presence of correlated FF inhibition, the unstimulated MSNs were less inhibited over the whole range of 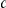 (Fig. 5D), leading to a small but significant reduction in the SNR (Fig. 5E). These results illustrate that, similar to the uncorrelated excitatory inputs, uncorrelated FF inhibition is optimal for signal representation in the striatum, though not as critical as the excitatory inputs. In the above we studied the effect of precisely coincident input spikes. However, in a biologically realistic scenario, spikes across different inputs may be jittered within a few ms. Our results are robust to such jittering of input spikes, except that the peak in Figs. 2D and 3D would become broader and shift to higher value of 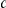 (data not shown).

### Experimental validation of the model

In spite of its simplicity, our network model can be validated using simultaneously recorded multiple single-unit spiking activity, routinely recorded in awake behaving animals. Whether (and to what extent) striatum neurons are driven by common inputs can be tested by either measuring spike correlations (or population synchrony, Fig. 3F), membrane potential correlations (Fig. 4D), or membrane potential fluctuation size among neurons that increase their firing rates in a behavioral task. Furthermore, the change in the correlation pattern (rather than in the firing rates) of the unstimulated neurons may provide additional information on the effective value of shared input correlations (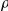; compare Figs. 2G and 3G). An experimental estimate of input correlations could validate our model and establish the importance of the spatio-temporal structure of cortical inputs in striatum network function. Simultaneous recording of single unit activities from 10–20 MSNs that modulate (increase/decrease) their activity in response to a behavioral task would be sufficient to obtain a reasonable estimate of the correlation structure in the striatum necessary to validate our model.

## Discussion

The striatum as the main input stage to the basal ganglia is involved in a variety of motor and cognitive functions. Anatomical studies and electrophysiological recordings in different behavioral conditions have provided useful hints regarding the information processing taking place in the striatum and the potential relevance of the structure of the cortico-striatal afferents. Previously, spiking network models of the striatum with randomly connected point neurons have been studied to understand the role of recurrent inhibition on network dynamics [40], [41] and assembly formation due to winner-less-competition [41]. Other network models were used to study the effect of dopamine on the formation of cell assemblies [42]. The properties of feedforward inhibition shaped by gap junctions were studied using networks with both reduced and detailed multi-compartment models [39], [43]. More recently, Humphries et al. [42] have integrated various levels of details such as distance-dependent connectivity among MSNs, and more realistic neuron and dopamine interaction models into a single striatum network. These various models have provided important insights into the computational role of various components of the striatum circuitry.

Beyond the local network structure, the organization of the afferents and efferents may also provide additional important insights into the functioning of a system. Therefore, here, we investigated the role of input correlations on striatum function which, to the best of our knowledge, has not been examined in a computational model before. Specifically, we addressed the question: how different types of input correlations affect the representation of cortical activity in the striatum. We showed that in a minimal network model of the striatum, there exists a preferred range of input correlation 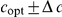 which enhances the representation of the cortical input, consistent with previous suggestions that striatum may be functioning as a correlation detector [44]. However, when striatal neurons shared their inputs (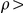
0), the SNR was reduced. This suggests that, given the network architecture of the striatum, there is a preferred cortico-striatal input configuration for optimal signal representation in the striatum: here, striatal neurons receive independent inputs and presynaptic pools of individual neurons have weak internal correlations. In addition, we also found that the signal representation of such input is optimal when the feedforward inhibition is uncorrelated.

Taken together, the absence of correlations among both excitatory and inhibitory inputs provides better signal representation in the striatal network. This effect of input correlations is a consequence of network-level interactions among the MSNs.

### Correlation structure of cortico-striatal inputs

In our model, the best SNR for the striatal representation of cortical input was obtained for shared correlation 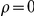, that is, for zero correlation among the input pools of the stimulated neurons. This requires, in terms of anatomy, no sharing of inputs among striatal neurons and, in terms of spiking activity statistics, no correlation between input pools of different striatal neurons. On the other hand, the best signal representation scenario for the striatum also required an optimum internal correlation 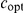 within individual input pools. It would appear to be a quite strict requirement to have 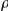 to be close to zero. However, anatomical evidence on the structure of the cortico-striatal projections suggests that 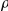 may indeed be very small within a local region.

Kincaid et al. (1998) suggested that neighboring MSNs receive nearly unique inputs from the cortex. From their results, striatal neurons with totally overlapping dendritic volumes have few presynaptic cortical axons in common, while cortical cells with overlapping axons have few striatal target neurons in common. Subsequent findings [13] relaxed this claim when considering extended axonal arborizations, in which separate branches might innervate distinct dendritic trees. However, while a typical cortico-striatal axon innervates a large volume, it makes only sparse contacts with the MSNs, so the average connectivity is still small, estimated to be less than 1%. Therefore, neighboring MSNs are not likely to share their inputs. Moreover, recent experimental work suggests that average correlations among cortical neurons may indeed be small [45]. Thus, the redundancy of nearby striatal neurons in response to cortical input signals is minimal.

In addition, it is conceivable that synapses formed by axons arising from functionally correlated brain regions could be selectively strengthened over time [46]–[48]. This may contribute to obtaining a weak, but optimum internal correlation 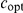 within the input pools to individual neurons.

### Stabilizing roles of FF and FB inhibitions

In our study we considered the possible scenario of correlated feedforward inhibition mediated by FSIs . We found that uncorrelated FSI activity is preferable to obtain a better signal representation in striatum. In this context, it is interesting that, to our knowledge, no correlated firing of FSIs has been observed *in vivo* [27].

We showed here that for a wide range of parameters within the biological range, the presence of both FF and FB inhibition actually does not cause synchrony or oscillation, unless the striatum is driven by such inputs. Moreover, it is known, and we have confirmed, that strong FB inhibition can lead to network oscillation, whereas strong FF inhibition can cause synchrony. Thus, we propose that the ongoing activity in the striatum of healthy animals is operating in an asynchronous low-rate activity regime, supported by a balance of the FF and FB inhibitions. The reason why shared input correlations reduce the SNR is that the stimulated neurons become correlated (Fig. 3D–H). Having an asynchronous background activity state in the striatum could reduce the correlation among the stimulated neurons and, thereby, improve the SNR.

It is possible that the balance of FF and FB inhibition is briefly disrupted during a behavioral task, and transient synchrony and/or oscillations may emerge [49]. Similarly, pathologies such as neuro-degeneration and dopamine depletion may also disturb the balance, thereby causing an increase in firing rates and associated synchrony. For instance, a deficit in FSIs has been observed in human patients with Tourette syndrome [50], which could lead to a reduction of FF inhibition. The motor tics observed in such patients may be related to the lack of inhibition in the striatal network [51].

### Implications for striatal function

**Figure 6:**
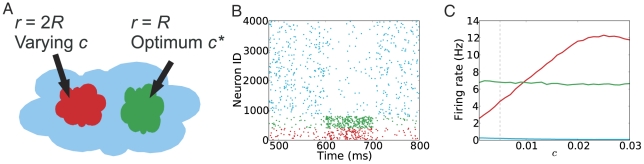
Both firing rate and input correlations affect the competition between two MSN groups. (A) A schematic of two competing functional groups of MSNs. The green group of MSNs received stimulus input (firing rate 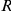 , correlations 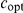). The red group of MSNs received stimulus input at a rate of 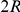, while the within-pool correlation was varied systematically. (B) Spiking activity in the MSNs when both red and green group received stimulus inputs. In this example within-pool correaltion for the the red group is suboptimal (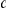 = 0.005). The green group has a higher output rate even though it receives a smaller amount of stimulated input. (C) The output rates of the two functional groups of MSNs when 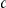 for the red group was varied systematically. The dashed line shows the value used in the panel B. When within-pool correlation for the red group is smaller than 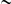
0.01, green group ‘wins’ even though it receives only half the input firing rate as compared to the red group, illustrating a function role that input correlation can play in the striatum.

Our findings indicate that higher input firing rates from the cortex alone do not guarantee a good signal-to-noise representation in the striatum. Instead, an appropriate combination of both higher rate and an optimum temporal correlation structure in the input determines the prominent representation in the striatum. Thus, information carried by weak inputs (low rates and/or correlations), presumably representing unfavorable choices, is screened out at the cortico-striatal interface. By contrast, signals corresponding to favorable choices (reflected in higher rates and/or correlations) may pass through this interface and be represented in the striatum. An illustrative example with two competing functional groups of MSNs is shown in Fig. 6A. Here, the green group receives a stimulus input with firing rate 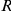 and within-pool correlation 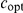. The red group, on the other hand receives twice the amount of stimulus input (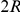). When the two groups compete, there is a regime when the within pool correlation for the red group is sub-optimal, the green group ‘wins’ even though it receives only half the amount of input (Fig. 6C). For this illustration, we considered the scenario of 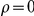 but non-zero 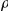 will lead to the same qualitative result.

The FB inhibition in the striatum has been reported to be weak, relatively sparse and with a fairly high failure rate, disqualifying it to support a winner-take-all dynamics. Alternatively, our findings suggest that the striatal recurrent inhibitory network can sharpen the contrast between the signal and the background noise (or weaker signals) by increasing the SNR. Moreover, the strong FF inhibition can further increase the contrast by constraining the overall activity in the network. Action selection processes presumably do not end in the striatum, but proceed in the downstream nuclei of the basal ganglia. Thus, the potential “winner” in action selection is unlikely to be determined already in the striatum stage. Yet, under the scheme proposed here, more favorable options, such as those receiving stronger and optimally correlated inputs, obtain a better representation in the striatum. It has been observed that different stimulus-reward contingencies are encoded in different fractions of striatal neurons responding [52]. From our simulation results with static synapses, a reduction in the number of activated MSNs could imply a drop in the performance of the signal representation. On the other hand, it has been reported that the number of striatal neurons responding to a task decreases during learning [2], [28]. As it may be expected that information becomes more reliably encoded in the course of the learning process, this might explain why fewer neurons need to be recruited to encode the same information, for instance because a more efficient signal representation scheme gradually takes over. However, more experimental data is needed to fruitfully address such issues within the the scope of our modeling work.

Striatal MSNs can be broadly subdivided into two classes, predominantly expressing either D1 or D2-type receptors which project to the direct and indirect pathways of the basal ganglia, respectively [53]. These two types of MSNs have different membrane properties and dendritic arbors [54]. As we have noted earlier, passive properties can determine the exact value of the optimal input correlation 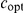. Likewise, the extent of dendritic arbors may alter the amount of input sharing (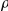) in the two types of MSNs. In view of the above, it is conceivable that these different properties of the D1 and D2 MSNs may specialize the direct and indirect pathways in terms of their optimal input correlations.

The robustness of our results depends crucially on the fact that the efficacy of correlated excitatory inputs (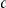) in generating a spike in the postsynaptic neuron changes in a first rising and then decaying fashion (Fig. 2D). This non-monotonic behavior is not affected in any qualitative manner by the time constant or the synaptic strength. For more detailed explanations we refer to our earlier work [35], [55]. Likewise, we find that the SNR of cortical inputs to the striatum decreases monotonically with the shared input correlation 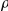 (Fig. 3H), because correlated inhibition leads to wasting of inhibitory inputs. This result depends on the temporal correlation of the inhibition, but not the exact values of synaptic time constants. For the reasons explained above, we only expect quantitative but not qualitative changes upon varying these and other parameters within the biological range.

In summary, we showed that for the network architecture of the striatum and the interplay of feedback and feedforward inhibitions, there is a preferred cortico-striatal input configuration for optimal signal representation in the striatum, which is a network phenomenon. The importance of input correlations is not restricted to signal representation in an inhibitory network (such as the striatum) alone. More generic neural networks with both excitatory and inhibitory neurons (such as the neocortex) may also exploit the structure of input correlations to modulate their response, both in output rates and correlations.

## Methods

### Models

#### Neurons

We considered two types of striatal neurons in our network model: medium spiny neurons (MSN) and fast spiking interneurons (FSI), both of which receive massive inputs from the cortex. The neurons in the network were modeled as leaky-integrate-and-fire (LIF) neurons, with subthreshold dynamics of the membrane potential 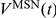 of a MSN 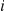 described by
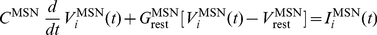
(1)where 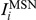 is the total synaptic input current to the neuron and 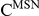, 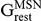 and 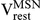 reflect the passive cell properties: capacitance, conductance at rest, and resting membrane potential, respectively. When the membrane potential reached a fixed spiking threshold 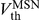 above resting potential, a spike was emitted. Then, the membrane potential was reset to its resting value and a pause for synaptic integration was imposed to mimic the refractory period in real neurons.

The subthreshold dynamics of the membrane potential 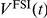 of a FSI 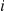 was described similarly by
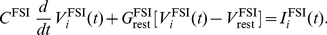
(2)The initial membrane potentials of both MSNs and FSIs were chosen from a uniform distribution (from −80 to −55 mV) to avoid any unwanted synchrony, caused by the initial conditions in the simulation runs.

#### Striatum network

A scheme of the striatal network model is shown in Fig. 1A. We simulated a network of two types of GABAergic neurons, 4,000 MSNs and 80 FSIs, according to the ratio given in the literature [56], [57]. Both types of neurons received independent excitatory Poisson inputs, mimicking the background cortico-striatal inputs. MSNs connect to other MSNs with a connection probability of 10% [58]. Each MSN received inhibitory inputs from 4–27 FSIs [59], therefore here we used an average value of 15, resulting in a 19% connectivity from FSIs to MSNs. In the striatum, FSIs are interconnected by gap junctions. However, *in vivo* recordings showed little correlation between nearby FSIs [27]. Moreover, a computational study found that synchronization effects due to gap junctions were moderate [39]. To this end, we investigated the impacts of both correlated and uncorrelated FSIs activity on our network model and its effects on signal representation. Two further cell types in the striatum, tonically active neurons (TANs) and dopaminergic neurons (DA), were not modeled explicitly in the network, but their modulatory effects on network connections were taken into account by changing the effective strengths of the FF and FB inhibitions, based on healthy animals' data [27], [28].

#### Synapses

Excitatory synaptic input was modeled by transient conductance changes using the alpha-function such that
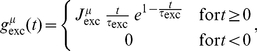
(3)where 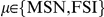 and the rise times for the excitatory synaptic inputs 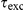 to the two neuron types were set to be identical. In addition, the MSNs were innervated by both feedforward (FF) inhibition from the FSIs and feedback (FB) inhibition from the MSNs. The corresponding conductance changes were
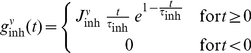
(4)where 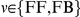. All inhibitory synaptic conductance transients were set to have identical rise times 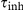. The peak amplitude 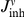 of the conductance transient was taken as the ‘strength’ of the synapse. By assuming fixed synaptic couplings, the total excitatory conductance 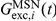 in a MSN 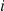 was given by
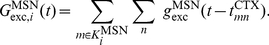
(5)The outer sum ran over all excitatory synapses 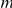 in the set 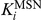 projecting onto neuron 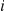, while the inner sum ran over the sequence of spikes (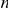
's) impinging on a particular synapse 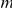. The set 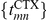 represents the spike times of the excitatory neuron 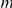.

Similarly, the total inhibitory conductance 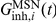 in an MSN 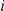 due to the FF and FB inhibitions was given by
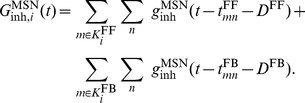
(6)

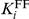
 and 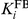 were the sets of presynaptic FSIs and MSNs projecting to MSN 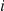, mediating the FF and FB inhibitions, respectively. Fixed transmission delays 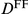 (1 ms) and 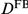 (2 ms) were imposed for the two inhibitions in all simulations. The total synaptic current into a MSN 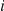 was
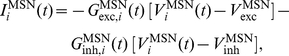
(7)with 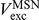 and 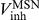 denoting the reversal potentials of the excitatory and inhibitory synaptic currents, respectively.

Similarly, the excitatory conductance 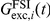 in a FSI 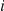 was given by
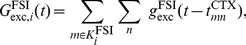
(8)and the total synaptic current into a FSI 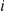 was
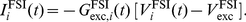
(9)The parameter values for both MSNs and FSIs in our network model are summarized in Table 1. The EPSP- and IPSP-sizes depended on both the synaptic conductances (see Tables 2 and 3) and the instantaneous membrane potential value in the respective cell [55].

**Table 1:** Parameter values of the model neurons used in this study.

Quantity	MSN	FSI
Number of neurons	4000	80
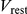 (mV)	−80 [69]	−80 [69], [70]
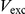 (mV)	0	0
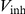 (mV)	−64 [9]	−76 [10]
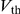 (mV)	−45 [59]	−54 [10]
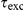 (ms)	0.3	0.3
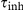 (ms)	2	2
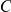 (pF)	200 [54]	500
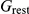 (nS)	12.5 [71]	25

**Table 2:** Specification of the cortical input to individual striatal neurons used in this study.

Target	Background input rate (Hz)	Peak conductance (nS)
MSN	2500	3.46
FSI	2500	5.5

**Table 3:** Specification of the two types of inhibition (FB, FF) within the striatum, used in this study.

Inhibition	Source	Target	Probability	Peak conductance (nS)	Delay (ms)
FB	MSN	MSN	0.1 [58]	0–0.5 (0.3 [9])	2.0
FF	FSI	MSN	0.19 [59]	0–2.5 (1.0 [59])	1.0

The values between the brackets were used in the simulations of the signal representation.

We constrained the network parameters such that the firing rate of MSNs was 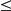
2 Hz in the resting state, and up to 25 Hz in the active state [27], [28]. FSI firing rates can vary from 5 to over 40 Hz, depending on the behavioral state of the animal [27], [49], [60]. Therefore, we set FSIs firing rates to 15 Hz and 20–40 Hz in the quiet wakefulness and active states, respectively. The synaptic conductance values we used are in the same range as estimated in another work [42].

In simultaneous intracellular recordings from interconnected cells, unitary inhibitory postsynaptic potentials (IPSPs) on MSNs were reported to be 1 mV and 0.3 mV at the soma for FF and FB inhibition, respectively [9], [59]. Based on these results, the corresponding strengths of inhibitory synapses for FF and FB inhibitions in our model, 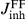 and 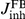, were set to 1 nS and 0.3 nS, respectively. In addition, in view of the fact that MSNs mainly project onto the dendrites of MSNs, whereas FSIs mainly project onto the soma, we used a larger delay for FB inhibition (2 ms), compared to the delay of 1 ms for FF inhibition.

We adopted the above values in all our network simulations, except in the Section on *Dynamic states of striatum network activity*, where we tuned the strengths of the connections over a range around the experimental values to explore and understand the individual effects of FF and FB inhibition on the network behavior. All network parameters used in our simulations are summarized in Tables 2 and 3. Results shown in Fig. 1 are based on simulation runs of 5 s for each parameter.

#### Correlated cortico-striatal inputs

Both neurophysiology and anatomy of cortico-striatal projections suggest that the inputs to the striatum may be correlated [14]–[18]. To model the cortical input to the striatum as correlated ensemble activity, we used the ‘multiple interaction process’ (MIP) [35] as the model input. In this process, correlated ensemble activity is generated by copying spikes from a common ‘mother’ Poisson process into 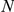 ‘children’ processes with a copying probability 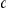, resulting in a population of 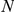 Poisson processes with pairwise correlation 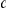.

In the learning stage of behaving animals, some MSNs responded with an increase in activity and others with a decrease [2], [37], [61], [62]. To mimic these observations, we stimulated a fraction of the striatal neurons (both MSNs and FSIs) with extra input on top of the background input; the size of the stimulated fraction was varied systematically from 10–40%, based on the reported experimental observations. The input firing rate refers to the ensemble activity. The choice of this stimulation range was to produce effects comparable to those observed in the experiments. The corresponding input spike trains to the stimulated cells were correlated based on MIPs (more details are provided below) and incorporated into the network via the Eqs. 5 and 8.

We systematically studied the effects of two kinds of input correlations on the network response: correlation between synaptic inputs onto individual neurons and input correlations among neurons. To this end we considered two different input configurations:

*Input configuration - I:* Individual neurons in the striatum received correlated input, however, inputs to different striatal neurons were uncorrelated. In the simplest scenario, this type of input means that neurons within the presynaptic pools of individual striatal neurons were correlated, but presynaptic pools of different striatal neurons were not correlated. In the simulations performed here, individual stimulated neurons each received 1,000 correlated spike trains with pairwise correlation 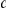 from a MIP pool, which was itself independent from all other presynaptic pools (Fig. 2A). Each stimulated neuron received a total of 200–400 Hz from the 1,000 spikes trains, in addition to the background activity.

*Input configuration - II:* Individual neurons in the striatum received correlated inputs; in addition, we allowed for correlations among the inputs to different striatal neurons. In the simplest scenario, this type of input means that the presynaptic pools of individual striatal neurons were either correlated or actually shared. To introduce correlations among the presynaptic pools of striatal neurons, we correlated the ‘mother process’ by a factor 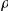. Using the MIP process to generate ‘children processes’, this resulted in a pairwise correlation of 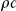 between spike trains from different presynaptic pools, while the correlation within a presynaptic pool remained unaffected, i.e. a correlation 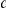 (Fig. 3A). Thus, 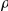 = 0 corresponded to input configuration - I, whereas 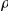 = 1 corresponded to the case that all input spikes trains to be used for stimulation were drawn from the same MIP, with pairwise correlation 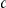. In the input configuration - II each stimulated neuron received a total of 400 Hz from the 1,000 spikes trains in addition to the background activity.

For each parameter set used in the input configurations I and II, we stimulated the striatal neurons for 100 ms, consistent with the epochs of transient rate increase observed in striatal activity in behavioral experiments [28], [37], [52]. To estimate the saliency of the signal representation (cf. Data analysis) we averaged the striatal responses over 50 (configuration I) and 150 (configuration II) trials, respectively. More trials were needed to obtain the statistical average in configuration II, because here, stimulated neurons received a higher proportion of shared input for larger 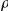 and, therefore, their responses were more variable.

#### Correlated feedforward inhibition

Fast spiking interneurons are known to be inter-connected by both chemical synapses and gap junctions. In general, gap junctions in neuronal networks can induce synchrony [38]. While there is no strong evidence for synchronization of striatal FSIs due to gap junctions, neither from experiments [27], nor from modeling studies [39], it is nevertheless of interest to study how correlated feedforward inhibition would influence the signal representation in the striatal projection neurons, as such correlation may arise as a function of cognitive state.

To this end, we modeled the spike trains of unstimulated FSIs with pairwise correlation 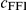 using the MIP process, whereas the stimulated FSIs were treated in the same way as before, i.e. they received correlated inputs from the cortex. The parameter 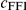 was varied systematically to assess the effect of correlated FSI activity.

All simulations were carried out using a Python interface to NEST [63]. The dynamical equations were integrated at a fixed temporal resolution of 0.1 ms.

### Model limitations

Here, we used a minimal striatum network model representing a small volume of the striatum to investigate the role of input correlations in signal representation in the striatum network. Below we discuss to what extent the simplifications we have made might influence our main results.

We described the effects of FF and FB inhibition and the signal representation in a reduced and simplified spiking network model of striatum. In addition to MSNs and FSIs, at least two other types of interneurons have been described in the striatum. The effects of the tonically active neurons (TANs) was incorporated implicitly into our model by modulating the strength of FF and FB inhibitions. Persistent low threshold spiking (PLTS) neurons are also known to inhibit the MSNs, but their output is relatively weak and sparse [64] and inclusion of the inhibitory effects of these neurons would not affect our conclusions qualitatively. Furthermore, the exact dynamics of cortico-striatal synapses was not included. The inclusion of slower synapses (e.g. NMDA type) or activity-dependent depression and facilitation of synaptic efficacy [65] would not cause a qualitative change to our results, as our main findings depend on the fact that the output firing rate is a non-monotonic function of the input correlations. This non-monotonicity arises due to the wasting of spikes which occurs when the size of the cluster of correlated events exceeds the amount required to reach spiking threshold. Thus, this behavior is independent of the choice of the synapse model: changing AMPA synapses to slower NMDA synapses may change the value of the optimum correlation (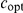), but it will not affect the non-monotonic behavior of the neuron and, hence, will not change our results qualitatively.

We emphasize that our choice of simple models for both single neurons and network topology was motivated by the fact that in such minimal setting we should be able to extract the most basic properties of the network. For instance, the issue how highly nonlinear membrane properties [66] might influence the representation of cortical inputs in the striatum is a complicated issue, which deserves a separate and more systematic analysis. It is worth mentioning that our results remained qualitatively unchanged when we replaced the simple integrate-and-fire neuron with a non-linear neuron model, namely, the adaptive exponential integrate-and-fire (AEIF) neuron [67] (data not shown).

In addition, we assumed that the 4,000 MSNs in our network model constitute only a small volume of striatum and, therefore, it is reasonable to assume a distance-independent random connectivity in the network.

### Data analysis

#### Network activity

The principal neurons, MSNs, are the only output neurons of the striatum. Thus, their dynamics are vital for the activity of the downstream nuclei of the basal ganglia. Therefore, to characterize the dynamical states of the striatum network, we focussed on analyzing the spiking activity of the MSNs. We used the following descriptors to characterize the network activity states:

*Firing rate* was estimated as the mean spike count per second of the MSN population in the network.

*Synchrony index* in the network was measured by the Fano factor, that is, the rate-scaled variance of the MSN population spike count 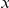:
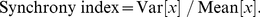
(10)where 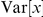 and 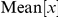 denote the variance and mean of the spike counts of the MSN population. To obtain a good estimate of the population spike counts, we recorded the spike trains of all neurons in the network and time binned (binwidth = 5 ms) their cumulative activity. A population of independent Poisson processes yields a synchrony index equal to 1, any mutual synchronization (correlation) results in an increase of 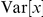 and, hence, in the synchrony index becoming larger than 1.

*Signal-to-Noise Ratio (SNR)* was computed as the ratio of the mean firing rates of stimulated MSNs and unstimulated MSNs. The SNR was used to measure the quality of signal representation in the striatal network activity, that is, the saliency of the response as compared to background activity. Interestingly, this saliency of the striatal response could form the basis for action-selection [68].

Because we were also interested in how the different input scenarios reflected on the subthreshold activity of the stimulated neurons (cf. Fig. 4), we performed additional simulations in which we recorded the *free membrane potential* (i.e. the membrane potential without spiking) [55] from selected neurons in the network. To this end, we ‘cloned’ them (letting them receive the same input as their respective twins) and switched off spiking in the clones.
